# Diagnostic Role of Bronchoalveolar Lavage in Patients with Suspected SARS-CoV-2 Pneumonia and Negative Upper Respiratory Tract Swab: A Systematic Review and Meta-Analysis

**DOI:** 10.3390/jcm11164656

**Published:** 2022-08-09

**Authors:** Federico Mei, Matteo Rota, Martina Bonifazi, Lina Zuccatosta, Francesco Porcarelli, Michele Sediari, Francesca Gonnelli, Alessandro Di Marco Berardino, Stefano Gasparini

**Affiliations:** 1 Department of Biomedical Sciences and Public Health, Marche Polytechnic University, Via Tronto 10/a, 60126 Ancona, Italy; 2 Respiratory Diseases Unit, Azienda Ospedaliero-Universitaria “Ospedali Riuniti”, 60126 Ancona, Italy; 3 Department of Molecular and Translational Medicine, Università degli Studi di Brescia, 25123 Brescia, Italy

**Keywords:** bronchoalveolar lavage, SARS-CoV-2 pneumonia, diagnostic yield, negative upper respiratory tract swabs

## Abstract

The added role of bronchoalveolar lavage (BAL) in SARS-CoV-2 detection in hospitalized patients with suspected COVID-19 pneumonia and at least one negative nasopharyngeal swab (NPS) has yet to be definitively established. We aimed to provide a systematic review and meta-analysis to summarize data from the literature on the diagnostic yield of BAL in this context. We searched Medline and Embase for all studies reporting outcomes of interest published up to October 2021. Two authors reviewed all titles/abstracts and retrieved the selected full texts according to predefined selection criteria. The summary estimate was derived using the random-effects model. Thirteen original studies, involving 868 patients, were included. The summary estimate of proportions of SARS-CoV-2 positivity in BAL fluid in patients with at least one previous negative NPS was 20% (95% confidence interval [CI]; 11–30%). Moreover, microbiological tests of BAL fluid led to the identification of other pathogens, mainly bacteria, in up to two-thirds of cases. BAL plays a crucial role in the diagnostic work-up of patients with clinical suspicion of COVID-19 and previous negative NPS, as it allowed to detect the infection in a significant proportion of subjects, who would have been otherwise misclassified, with relevant implications in the prevention of disease spread, especially in hospital settings.

## 1. Introduction

Since the beginning of the COVID-19 pandemic, SARS-CoV-2 nucleic acid detection by real-time reverse-transcriptase polymerase chain reaction (RT-PCR) in nasopharyngeal swabs (NPS) has been considered a reference standard for a definite diagnosis. However, a significant proportion of false-negative cases has been reported in the literature, [1] as test performance might be influenced by several factors, including selected “intrinsic” patient characteristics (i.e., stage of disease, viral load), the prevalence of disease, as well as technical aspects in collecting and managing specimens. Therefore, in hospitalized patients with a high clinical suspicion of COVID-19 but a baseline negative NPS, strategies to increase diagnostic confidence, such as repeating the test or performing RT-PCR on other biological samples, have been proposed and integrated into daily practice with different algorithms, mainly determined by local sources and skills.

Bronchoalveolar lavage (BAL) is considered a valuable tool in this context, as it allows to perform RT-PCR on lung fluid as well as other microbiological tests, potentially helpful for a proper differential diagnosis [1]. The accuracy of BAL in SARS-CoV-2 detection has been assessed in a number of studies, showing good results overall, but the specific added value of this procedure in the subgroup of patients with suspected COVID-19 pneumonia and at least one negative NPS has yet to be definitely established, as conflicting evidence has emerged over time.

Considering that bronchoscopy is an aerosol-generating procedure, and it can worsen gas exchanges in patients with acute respiratory failure, it is of utmost importance to balance the risks of this technique with the benefits of identifying subjects in whom the diagnosis would have been missed otherwise, with relevant implications in the management and prevention of disease spreading, especially in hospital settings.

Therefore, the aim of the present systematic review and meta-analysis is to summarize data from the literature on the diagnostic yield of BAL for the detection of SARS-CoV-2 in cases with high clinical suspicion for COVID-19 and at least one negative NPS.

## 2. Materials and Methods

A systematic review of the literature was performed according to guidelines developed by the Meta-analysis Of Observational Studies in Epidemiology group [2]. We searched Medline and Embase for all original articles on BAL accuracy in the diagnostic work-up of COVID-19 published up to October 2021, using a combination of free text and MESH/Emtree terms related to COVID-19 diagnostic work-up and bronchoscopy (see Supplementary Materials). The electronic search was supplemented by hand-searching the bibliography of relevant articles [2].

The systematic review was registered at PROSPERO with the number CRD42021283450. 

We included all studies reporting data on the diagnostic yield of BAL for SARS-CoV-2 detection in patients with suspected COVID-19 and at least one negative NPS. 

Exclusion criteria were (1) studies reporting data on the diagnostic yield of BAL for SARS-CoV-2 detection in patients with positive NPS; (2) studies reporting data on the diagnostic yield of BAL for SARS-CoV-2 detection in patients without suspected COVID-19 (i.e., preoperative screening); (3) studies reporting data on the diagnostic yield of laryngotracheal aspiration for SARS-CoV-2 detection in patients with suspected COVID-19; (4) case-series of fewer than 20 patients; and (5) non-English language full-text articles.

### 2.1. Study Screening and Ascertainment of Eligibility

Two independent authors (F.P. and F.G.) reviewed all titles/abstracts and retrieved detailed full texts of potentially relevant articles. Disagreements were resolved by discussion. When multiple reports were available on the same cohort of patients, we included the most recent or informative one. 

The two reviewers independently retrieved information, and qualitative and quantitative data were collected in ad hoc electronic form. 

The following data were recorded for each study: The first author’s name, year of publishing, country, study design (prospective; retrospective), sample size, study population, number of negative NPSs, timing between negative NPSs and BAL (hours), qualitative radiological pattern at CT (ground glass opacity, consolidations, crazy paving), and additional pathogens detected at BAL microbiology.

The measure of interest was the proportion of subjects with SARS-CoV-2 detection at BAL.

### 2.2. Risk of Bias Assessment and Statistical Analysis 

We assessed the studies for methodological quality using the revised Quality Assessment of Diagnostic Accuracy Studies (QUADAS-2) tool. This consists of two sections, aimed to assess the risk of bias and applicability concerns using pre-defined key domains (patient selection, index test, reference standard, flow and timing of patients’ selection of the index tests and reference standard). Patient selection, the index test, and the reference standard are examined concerning the risk of bias and applicability concerns, while the flow and timing of patient selection address the risk of bias only. Each domain is rated as “low”, “high”, or “unclear” for both the risk of bias and concerns about applicability. If a study is judged as “low” on all domains relating to bias or applicability, then it receives an overall judgment of “low risk of bias” or “low concern regarding applicability”. If a study is judged “high” or “unclear” in 1 or more domains, then it may be judged “at risk of bias” or as having “concerns regarding applicability”.

The diagnostic yield of BAL for SARS-CoV-2 detection in patients with suspected COVID-19 and at least one negative NPS was summarized across studies in terms of proportion using the Freeman–Tukey Double arcsine transformation. As we anticipated heterogeneity across studies, the random-effects model with the DerSimonian and Laird estimator of the variance component was fitted [3]. Heterogeneity among studies was assessed using the χ^2^ test, defining significant heterogeneity as a *p*-value < 0.10, while inconsistency was quantified using the I-squared statistic. Meta-analytic results were summarized within forest plots. 

A leave-one-out study in a time-sensitivity analysis was carried out to assess the possible influential role of each included study on the pooled estimate.

Publication bias was assessed through visual examination of funnel plot asymmetry and tested using Egger’s test. 

Given the limited number of studies included in the meta-analysis, no stratified analyses were conducted.

All analyses were carried out with the “metafor” package.

## 3. Results

**Figure 1:**
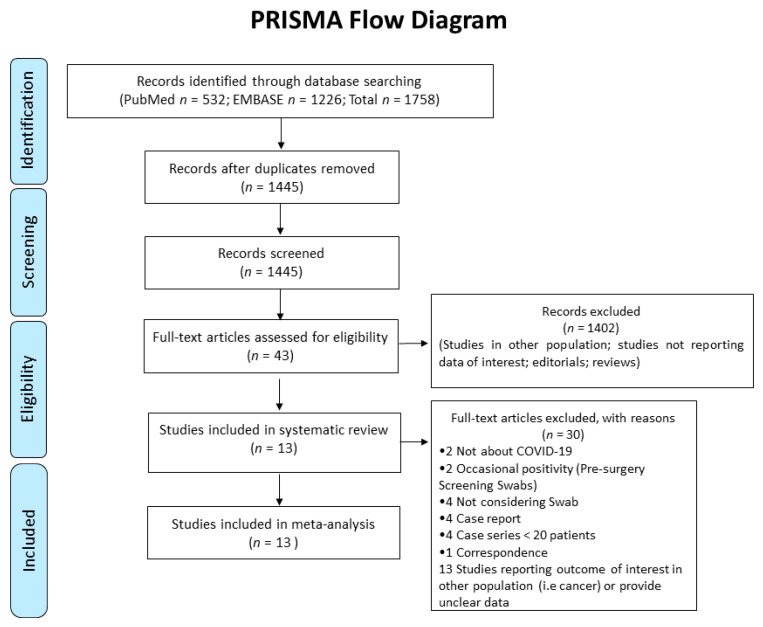
Flow-chart of study selection.

After removing duplicates between Medline and Embase, the systematic review identified 1445 references (shown in Figure 1). 

The initial screening based on title/abstracts led to the exclusion of 1402 papers, due to non-relevance to the topic. The remaining 43 articles were retrieved for detailed full-text evaluation, and thirty papers did not fulfil eligibility criteria. The main reasons for exclusion were the following: Studies not reporting data of interest, editorials, reviews, case reports, case-series with less than 20 patients included, studies reporting an outcome of interest but in other settings (i.e., pre-surgery screening in not suspected patients) or in other populations (i.e., patients with cancer), studies reporting an outcome of interest without specifying results from previous NPSs, studies reporting an outcome of interest without providing different results according to the positivity/negativity of previous NPSs.

Thus, 13 original studies were included in the present review for both qualitative and quantitative analyses. Their main characteristics are presented in Table 1. The majority of included studies were conducted in Italy (*n* = 7), followed by Belgium (*n* = 4), Spain (*n* = 1), and the US (*n* = 1). All investigations were observational studies, of which 11 were retrospective and 2 were prospective. The mean age of the study population was between the fifth and the sixth decade, and there was a male predominance in all studies reporting this information. The number of pre-BAL NPSs was heterogenic across investigations, ranging from 1 to 3, and all were performed within 72 h of the procedures. Consolidations and GGO, alone or in combination, were the most common patterns observed in the CT scan. Data on microbiology were available in eight studies and the rate of pathogen detection ranged from 9 to 61%, with bacteria being the most prevalent.

**Figure 2:**
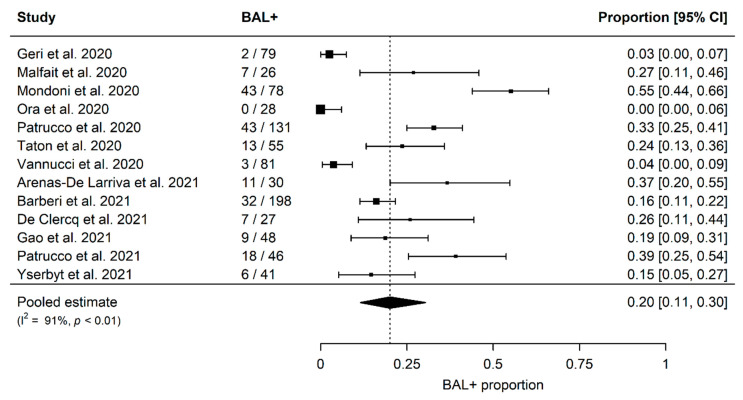
Overall proportion of SARS-CoV-2 positivity in BAL fluid [4,5,6,7,8,9,10,11,12,13,14,15,16].

The summary estimates of proportions of SARS-CoV-2 positivity in BAL fluid in patients with at least one previous negative NPS are shown in Figure 2. The overall proportion, derived from 13 studies including 868 patients, was 20% (95% confidence interval [CI]; 11–30%), and significant heterogeneity among studies was detected (I2 91%, *p* < 0.01) (Figure 2). 

**Figure 3:**
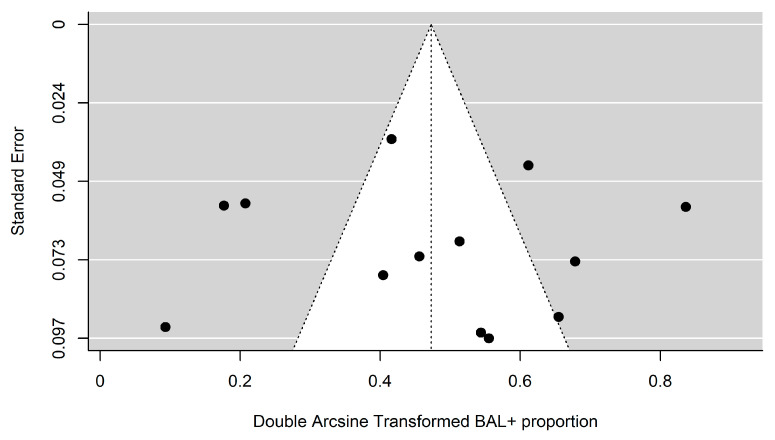
Contour-enhanced funnel plots for BAL.

The funnel plot revealed to evaluate publication bias is reported in Figure 3. Although the funnel plot (Figure 3) appears slightly asymmetric, Egger’s test results did not reveal evidence of a significant publication bias (*p* = 0.8833).

**Figure 4:**
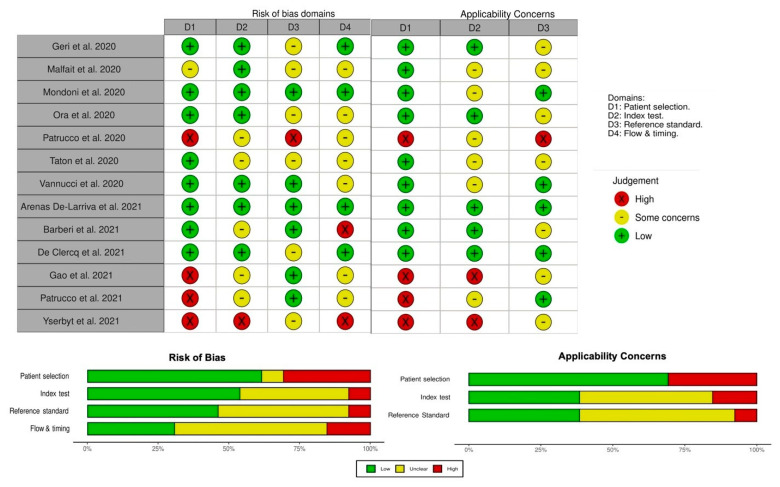
Graphical display of the revised Quality Assessment of Diagnostic Accuracy Studies (QUADAS-2) results, according to risk of bias and applicability concerns [4,5,6,7,8,9,10,11,12,13,14,15,16].

The application of the QUADAS-2 tool is summarized in Figure 4. Overall, two studies were judged as “low risk of bias”, two studies were deemed as having “low concerns regard applicability” to the review question, and only one study met both the conditions. The remaining were considered to have risk of bias and concerns about applicability (Figure 4). 

## 4. Discussion

The present systematic review and meta-analysis on the diagnostic performance of BAL in detecting SARS-CoV-2 RT-PCR positivity in patients with at least one previous negative NPS confirm the valuable role of this procedure, as overall it allowed diagnosis of the infection in a significant proportion of subjects, who would have been otherwise misclassified. Moreover, microbiological tests of BAL fluid led to the identification of other pathogens, mainly bacteria, in up to two-thirds of cases, providing, thus, useful information to guide proper management. 

The sensitivity of RT-PCR in different biological specimens is variable and depends on several factors [1], including the timing of the diagnostic test [17]. In the prodromal phase, when contagiousness is higher, the active viral replication of the virus can be better identified in the upper airways, but in later stages, when lung involvement is predominant, the viral load is likely to be more prevalent in the lower airways [17]. For instance, the Infectious Diseases Society of America Guidelines on Molecular Diagnostic Testing of COVID-19 underline the importance of the timing of specimen collection in the relationship with the onset of symptoms, and they suggest that, in the context of hospitalized cases with an initial negative NPS and high clinical suspicion, it is preferable to collect a lower respiratory tract sample rather than performing another upper respiratory sample [18].

Considering the relatively high proportion of a first negative NPS in daily practice, identifying patients with a higher likelihood of SARS-CoV-2 BAL positivity is crucial to reduce unnecessary procedures and optimize the risk–benefit profile of diagnostic work-up for patients and healthcare workers. A suggestive clinical scenario and/or specific CT characteristics are the main significant predictors of positive SARS-CoV-2 RT-PCR at BAL, and thus, statements of Interventional Pulmonology Experts recommend performing nonelective bronchoscopy in the presence of clinical or radiological suspicion as confirmation/exclusion of SARSCoV-2 infection in patients with previous non-diagnostic tests [19,20,21]. However, it is of utmost importance to also consider the concurrent epidemiological scenario, as it hugely influences the pre-test probability, and, thus, the choice of proper diagnostic test should always be based on its expected role on post-test probability, carefully balancing the risk–benefit profile. Moreover, in the case of very high pre-test probability, the lack of SARSCoV-2 detection in BAL fluid does not allow one to definitively exclude the infection, as false negative results, although rare, have also been described in this context [18,22].

With reference to safety, one of the main concerns about performing bronchoscopy in the COVID-19 pandemic was the supposed high risk of viral transmission to healthcare workers. However, robust evidence on the high risk of being infected during bronchoscopy with relative quantitative estimates is still lacking. Saha B.K. et al. summarized data from seven cohort studies that assessed the risk of COVID-19 transmission among bronchoscopists and other healthcare workers in a total of 650 mechanically ventilated patients [23]. Sixty bronchoscopists were involved with an average of 16.8 exams each, and only two of them were infected with SARS-CoV-2, while none of the bedside nurses, respiratory therapists, or technicians were [23]. Therefore, when personal protective equipment is appropriately used, the risk appears to be lower than expected. 

A major strength of the present study is that, although a number of previous meta-analyses on SARS-CoV-2 RT-PCR BAL sensitivity have been published over the last two years [24,25], this is the most recent one and was focused primarily on estimating the overall added value of this procedure in the challenging scenario of an initial negative NPS and high clinical suspicion. 

However, some limitations have to be underlined. First, significant heterogeneity among investigations was observed in terms of the sample size, the number and timing of previous NPSs, and the prevalence of disease, but the small number of studies included did not allow sensitivity analyses to explore the impact of these features. Moreover, in view of the absence of an international statement on the standardization of BAL execution, confounding factors could have also affected the performance of the index test, due to potential procedural differences in collecting lung fluid. Second, most investigations were not primarily designed as prospective studies, but they retrospectively reported experiences from routine clinical practice, likely affecting the methodological quality of data, as reported by QUADAS-2 results. Third, geographical coverage was very limited, as most studies were performed in Europe (mainly Italy and Belgium), one in the US, and none in Asian countries.

## 5. Conclusions

In conclusion, the present synthesis of the literature strongly supports the key role of BAL in the diagnostic work-up of patients with high clinical suspicion of COVID-19 and a negative NPS, as it allowed for the detection of infection in one out of five subjects, thus, significantly reducing the proportion of false-negative cases, with subsequent relevant implications in the management and prevention of disease spreading, especially in hospital settings. 

## Figures and Tables

**Table 1:** Characteristics of included studies.

Author/Year	Country	Study Design	Sample Size	Age (Mean ± SD)	N° of NEGATIVE Pre-BAL Swab (N° Pts)	Timing of NEGATIVE Pre-BAL Swab (Hours)	Chest CT Pattern	Additional Pathogens Rate in Overall BAL
**Geri et al. [4]**	Italy	Retro	79	65 ± 17	1 (*n* = 29) 2 (*n* = 46) 3 (*n* = 4)	36	CP and GGO (80%)	N/A
**Malfait et al. [5]**	Belgium	Retro	26	N/A	1	N/A	N/A	N/A
**Mondoni et al. [6]**	Italy	Retro	78	60 ± 13	2	N/A	N/A	4/43 (9%) (Bacteria = 1; Fungi = 3)
**Ora et al. [7]**	Italy	Retro	28	65 ± 16	3	N/A	GGO (68%) Cons (32%)	13/28 (46%) (Bacteria = 6; Fungi = 7)
**Patrucco et al. [8]**	Italy	Retro	131	64 ± 14	1 (*n* = 11) 2 (*n* = 120)	N/A	GGO (82%) Cons (58%)	46/131 (35%) (Bacteria = 30; Fungi = 19)
**Taton et al. [9]**	Belgium	Retro	55	62 ± 16	1 (*n* = 31) 2 (*n* = 24)	N/A	CP or GGO + Cons or Cons (53%)	24/55 (44%) (Bacteria = 15)
**Vannucci et al. [10]**	Italy	Retro	81	69 ± 16	2 (*n* = 53) 3 (*n* = 28)	within 72 h	GGO + Cons (100%)	24/81 (27%) (Bacteria = 16; Fungi = 8)
**Arenas-De Larriva et al. [11]**	Spain	Pros	30	59 ± 15	2	N/A	Cons (23%) *	N/A
**Barberi et al. [12]**	Italy	Retro	198	68 ± 14	1 (*n* = 185) 2 (*n* = 12) 3 (*n* = 1)	N/A	GGO (46%) Cons (33%)	N/A
**De Clercq et al. [13]**	Belgium	Pros	27	56 ± 13	2	N/A	GGO (100%) Cons (92%)	14/27 (52%) (Bacteria = 7)
**Gao et al. [14]**	US	Retro	48	N/A	1	24	N/A	17/78 (22%) (Bacteria = 17)
**Patrucco et al. [15]**	Italy	Retro	46	61 ± 15	2	36	GGO + Cons (32%)	N/A
**Yserbyt et al. [16]**	Belgium	Retro	41	N/A	1	N/A	GGO + Cons (20%)	25/41 (61%) (Bacteria = 13; virus = 9; Fungi: 8)

Footnotes: BAL = Broncho-alveolar lavage; CP = crazy paving; CT = computed tomography; GGO = ground glass opacities; * it refers to Chest X-ray.

## Data Availability

Not applicable.

## Supplementary Materials

The following supporting information can be downloaded at: https://www.mdpi.com/article/10.3390/jcm11164656/s1, Search strategy.
