# Supplementary material for: Diagnostic Role of Bronchoalveolar Lavage in Patients with Suspected SARS-CoV-2 Pneumonia and Negative Upper Respiratory Tract Swab: A Systematic Review and Meta-Analysis
Source: J Clin Med. 2022 Aug 9;11(16):4656. doi: 10.3390/jcm11164656 (PMC9409822; doi:10.3390/jcm11164656)
Supplement: Supplementary file 1 [file jcm-11-04656-s001.zip › jcm-1830613-supplementary.pdf]

Supplementary materials

Search strategy

PUBMED:
("bronchoalveolar lavage"[MeSH Terms] OR ("bronchoalveolar"[All Fields] AND "lavage"[All Fields])
OR "bronchoalveolar lavage"[All Fields] OR ("bronchoalveolar lavage fluid"[MeSH Terms] OR ("bronchoalveolar"[All Fields]
AND "lavage"[All Fields] AND "fluid"[All Fields]) OR "bronchoalveolar lavage fluid"[All Fields])
OR ("bronchoscopy"[MeSH Terms] OR "bronchoscopy"[All Fields] OR "bronchoscopies"[All Fields]))
AND ("covid 19"[All Fields] OR "covid 19"[MeSH Terms] OR "covid 19 vaccines"[All Fields] OR "covid 19 vaccines"[MeSH Terms]
OR "covid 19 serotherapy"[All Fields] OR "covid 19 serotherapy"[Supplementary Concept]
OR "covid 19 nucleic acid testing"[All Fields] OR "covid 19 nucleic acid testing"[MeSH Terms]
OR "covid 19 serological testing"[All Fields] OR "covid 19 serological testing"[MeSH Terms]
OR "covid 19 testing"[All Fields] OR "covid 19 testing"[MeSH Terms] OR "sars cov 2"[All Fields]
OR "sars cov 2"[MeSH Terms] OR "severe acute respiratory syndrome coronavirus 2"[All Fields]
OR "ncov"[All Fields] OR "2019 ncov"[All Fields]
OR (("coronavirus"[MeSH Terms] OR "coronavirus"[All Fields] OR "cov"[All Fields])
AND 2019/11/01:3000/12/31[Date - Publication]))

EMBASE
'lung lavage'/exp OR 'bronchoalveolar lavage fluid'/exp OR 'bronchoscopy'
AND 'coronavirus disease 2019'/exp
